# There is no belief update bias for neutral events: failure to replicate Burton et al. (2022)

**DOI:** 10.1080/20445911.2023.2245112

**Published:** 2023-08-14

**Authors:** Neil Garrett, Tali Sharot

**Affiliations:** a School of Psychology, University of East Anglia, Norwich, UK; b Affective Brain Lab, Department of Experimental Psychology, University College London, London, UK; c The Max Planck UCL Centre for Computational Psychiatry and Ageing Research, University College London, London, UK; d Department of Brain and Cognitive Sciences, Massachusetts Institute of Technology, Cambridge, MA, USA

**Keywords:** Optimism, belief updating, asymmetric learning

## Abstract

In a recent paper, Burton et al. claim that individuals update beliefs to a greater extent when learning an event is less likely compared to more likely than expected. Here, we investigate Burton’s et al.’s, findings. First, we show how Burton et al.’s data do not in fact support a belief update bias for neutral events. Next, in an attempt to replicate their findings, we collect a new data set employing the original belief update task design, but with neutral events. A belief update bias for neutral events is not observed. Finally, we highlight the statistical errors and confounds in Burton et al.’s design and analysis. This includes mis-specifying a reinforcement learning approach to model the data and failing to follow standard computational model fitting sanity checks such as parameter recovery, model comparison and out of sample prediction. Together, the results find little evidence for biased updating for neutral events.

## Introduction

Unrealistic optimism (Weinstein, 1980) is the tendency to underestimate the likelihood of negative events (such as illness, accident and natural disasters) and overestimate the likelihood of experiencing positive events such as professional success (Wiswall & Zafar, 2015). It impacts human decision making in domains ranging from politics to medical care (Krieger et al., 2016; Paling, 2003; Staats et al., 2018). Understanding the mechanism that generates unrealistic optimism is crucial for developing methods to mitigate this bias, for example in relation to beliefs about the risk of climate change (Sunstein et al., 2016) or COVID-19 (Globig et al., 2022).

A core mechanism shown to generate and maintain positive beliefs is asymmetric belief updating (Benjamin, 2019; Eil & Rao, 2011; Kappes et al., 2018; Korn et al., 2012; Kube & Rozenkrantz, 2021; Kuzmanovic et al., 2018, 2015; Mobius et al., 2011) whereby individuals place a larger weight on information that is better than expected, compared to worse than expected, when updating their beliefs. This phenomenon has been shown both in relation to beliefs about future negative events (e.g. learning that the likelihood of being a victim of card fraud is lower than expected leads to greater belief updating than learning it is higher than expected) and positive events (e.g. learning that the likelihood of winning a grant is higher than expected leads to greater belief updating than learning it is lower than expected). This asymmetry in updating can lead to optimistic beliefs, biased self-perceptions and flawed financial predictions (Barber & Odean, 1999; Dunning et al., 2004; Eil & Rao, 2011; Greenwald, 1980; Kube et al., 2022; Kube & Rozenkrantz, 2021; Kuhnen, 2015; Peysakhovich & Karmarkar, 2016; Russo et al., 1996; Shefrin, 2009).

All past studies (Kappes et al., 2018; Kube et al., 2022; Kuzmanovic et al., 2018, 2016, 2015; Kuzmanovic & Rigoux, 2017; Ma et al., 2016; Oganian et al., 2019; Sharot et al., 2011) except one (Burton et al., 2022) have examined optimistic biased updating in relation to beliefs about future aversive (e.g. catching a cold) or positive (e.g. being invited to a party) events. For negative events, beliefs are updated more in response to learning the likelihood of the negative event is *lower* than expected (Garrett & Sharot, 2017; Kappes et al., 2018; Kuzmanovic et al., 2018, 2016, 2015; Kuzmanovic & Rigoux, 2017; Oganian et al., 2019; Sharot et al., 2011). For positive events, beliefs are updated more in response to learning the likelihood of the positive event is *greater* than expected (Garrett & Sharot, 2017). Recently, Burton et al., examine belief updating for neutral events. They loosely base their study on the belief update task (Kappes et al., 2018; Kuzmanovic et al., 2018, 2016, 2015; Kuzmanovic & Rigoux, 2017; Ma et al., 2016; Oganian et al., 2019; Sharot et al., 2011; Sharot & Garrett, 2022). They claim to show a greater degree of updating after learning that neutral events are less likely than expected compared to more likely. They suggest that their results implies that the optimistic update bias found when examining updating for positive and/or negative events is not genuine.

However, instead of using the classic task (Oganian et al., 2019; Ossola et al., 2020; Sharot et al., 2011) to investigate this, Burton et al. alter the task, changing the response scale and the distribution of probability base rates, among other modifications. These modifications, as discussed in detail below, have been previously documented to introduce confounds, not present in the original task, which will indeed lead to false positives (Garrett & Sharot, 2017; Sharot & Garrett, 2022).

Here, we attempted to replicate Burton et al.’s findings of biased belief updating with neutral stimuli. We were unable to replicate their results when analysing Burton et al.’s own data. In addition, we found evidence that their implementation of the task introduced confounds in the design and analysis. Moreover, we were unable to replicate their results in a new data set we collected (using an unconfounded set of stimuli), finding no evidence of bias. Together, these findings suggest that there is no evidence for asymmetric belief updating for neutral events.

## Materials and methods

### Participants

One hundred participants were recruited via the online platform Prolific. This sample size is the same as the one used by Burton et al., in Experiments 1–3. Completion of the experiment took approximately 1 hour and participants were compensated for their time. The study was approved by UCL’s Ethics Committee. This study was not preregistered.

### Task

**Figure 1.**
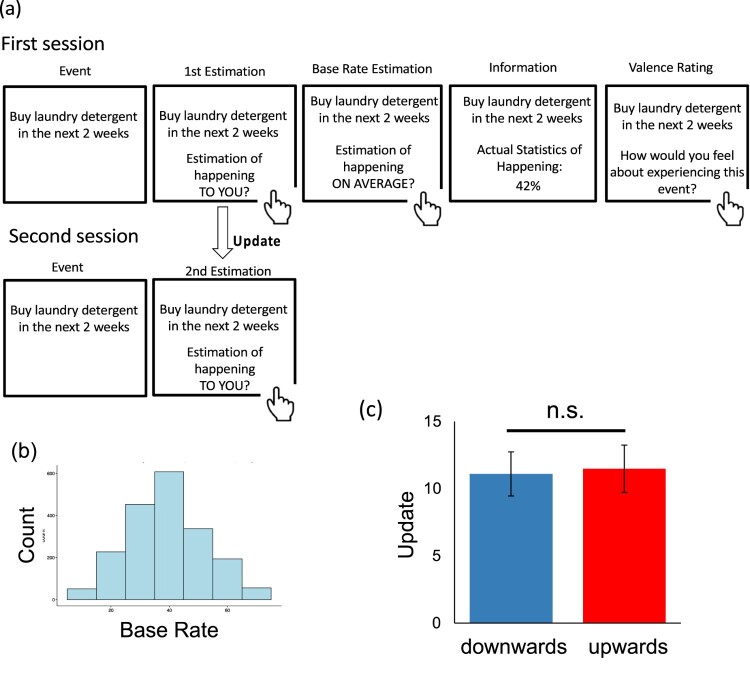
Task design. (a) On each trial, participants (*N* = 100) were presented with a short description of 1–39 events and asked to estimate how likely this event was to occur to them. Estimates were entered into a text box displayed on the computer screen using a computer keyboard on a scale between 3% and 77%. Participants were then asked to estimate how likely the event was to occur on average in the population on the same scale. They were then presented with the average probability of that event occurring to a person like themselves (derived from factual sources, see Supplementary Materials). In a second session, participants were asked to re-estimate how likely the event was to occur to them. For each event, an update term was calculated as the difference between the participant’s first and second estimations, such that positive numbers indicate a move towards the base rate. (b) All events probabilities lay between 10% and 70% with a midpoint of 40. (c) Following Burton et al. (2022), we plot the magnitude of belief updating for events rated as neutral by participants, predicted by the linear mixed effects model with bars representing 95% confidence intervals. As can be observed, there is no asymmetry in belief updating for trials in which participants learned the event is more likely than they had originally estimated (upwards) or less likely (downwards). In other words, we were unable to replicate the difference in updating Burton et al. report.

The study involved two sessions (Figure 1(a)). In a first session, each participant was presented with one of 39 life events (e.g. Buy laundry detergent in the next two weeks) and asked to imagine the event happening to them. They were then asked to estimate how likely that event was to happen to them (E1); participants were also asked to give a second estimate of the likelihood of the event happening to an average person in the population (eBR; an estimate of the base rate). Participants were instructed to type in each estimate between 3% and 77% and were not able to enter responses outside of this range. There were no restrictions on participants’ response time. The order of the two estimates (E1 and eBR) was counterbalanced between subjects by randomly assigning each participant to one of two conditions: E1 followed by eBR (*N* = 51), or eBR followed by E1 (*N* = 49). After these two initial estimates were recorded, participants were shown the base rate statistic (BR) of the event happening to someone from the same socioeconomic environment as them, which ranged from 10% to 70%. Finally, participants were asked to rate how negative or positive they found the event on a five-point scale (1 = very negative, 2 = negative, 3 = neural, 4 = positive, 5 = very positive). In a second session, which took place immediately after the first session, participants were asked to re-estimate how likely each event was to happen to them (E2). Again, there were no restrictions on participants’ response time.

After completion of the task, we tested participants’ memory for the information presented. Participants were asked to recall the information previously presented (BR) of each event. Subsequently, participants were then asked to rate all life events according to their past experience with each event (“Has this event happened to you before?” From 1 = never to 6 = very often), vividness of imagination (“How vividly could you imagine this event?” From 1 = not vivid to 6 = very vivid); familiarity (“Regardless if this event has happened to you before, how familiar do you feel it is to you from TV, friends, movies and so on?” From 1 = not at all familiar to 6 very familiar); and arousal (“When you imagine this event happening to you how emotionally arousing is the image in your mind?” From 1 = not arousing at all to 6 = very arousing). The survey was constructed and presented using web based survey service Qualtrics.

### Analysis

The aim of this study was to examine if a bias emerges in updating beliefs about neutral life events. Life events were categorised as neutral for each participant individually according to their own evaluation. Specifically, events were classified as neutral if the participant rated the event as 3 during the task (mean [sd] number of trials rated neutral per participant: 19.23 [6.56]) and we made sure to only include these neutral events in the analysis.

Participants could either receive information in a “downwards direction” or an “upwards direction” depending on whether the participant initially overestimated or underestimated the probability of the event relative to the base rate, respectively. Specifically, if their first estimate (E1) was higher than the base rate presented (BR), the information would be categorised as “downwards” and if their first estimate (E1) was lower than the base rate presented (BR), the information would be categorised as “upwards”. Trials in which the initial estimate was equal to the statistic presented were excluded from subsequent analyses as these could not be categorised into either condition. In addition, we followed the exclusion criterion employed by Burton et al. Specifically, mean updates in each of the two conditions (upwards/downwards) were calculated and outliers were removed (±3 × the interquartile range).

### Linear mixed effect models

Belief update was calculated for each trial and participant as the difference between first and second estimate. As done previously (e.g. Kuzmanovic et al., 2015) and followed by Burton et al., update was calculated such that positive scores indicate a move towards the base rate and negative scores a move away from the base rate:

$$\begin{array}{rl} & update\;(downwards)\;=\;E1\text{–}E2\\ & update\;(upwards)\;=\;E2\text{–}E1 \end{array}$$

Following Burton et al. we used a linear mixed effects (LMM) model with update entered as the dependent variable, direction of error (upwards/downwards) as a fixed factor, and participant as a random factor, including intercepts and slopes as random effects. In the syntax of the lme4 package, the specification for the regression was as follows:

update∼direction+(1+direction|Participant)

We then used Type III tests and Satterthwaite’s approximation for degrees of freedom to calculate the statistical significance of the fixed effects. We also examined whether we could detect an effect if we ran the LMM without random slopes, i.e.

update∼direction+(1|Participant)

To be clear, we do not think this is a valid model specification but wanted to test whether even with this very lenient approach that Burton et al. took to the data, a false positive could arise for neutral events when the proper experimental design was used.

Finally, we reran the LMM (with both random intercepts and slopes) excluding trials (25% of trials rated neutral) that would be assigned into a different category under and alternate classification scheme (Garrett & Sharot, 2017, 2014) in which trials were partitioned into downwards/upwards according to whether participants estimate of the base rate (eBR) was higher (downwards) or lower (upwards) than the base rate presented (BR).

### Linear regression

Next we examined the relationship between estimation errors and update. For each trial, an estimation error term was calculated as the unsigned difference between the probability presented and participants’ first estimate on that trial (the likelihood the event happens to them, i.e. E1).

$$\begin{array}{rl} estimation\;error\;=\\ & \left|probability\;presented-first\;estimate\;\right| \end{array}$$

We estimated the extent to which participants integrated new information into their beliefs by regressing absolute estimation errors against update scores separately for upwards and downwards trials for each participant:

$$\begin{array}{rl} & Update\;(downwards)\;=\;b0\;+\;b1*estimation\;error\\ & Update\;(upwards)\;=\;b0\;+\;b1*estimation\;error \end{array}$$

This resulted in two scores (the unstandardised regression coefficients b1 in the equations above) for each participant: one for upwards trials and one for downwards trials. These were compared with one another using paired sample *t* tests.

### Bayesian analysis

This analysis directly follows the procedure of Burton et al.

Participants estimate of each event occurring to themselves in the future (E1) and estimate of the base rate (eBR) were used to calculate an Implied likelihood Ratios (LHR) on each trial as:

$$LHR=\frac{E1}{1-E1}\;÷\frac{eBR}{1-eBR}$$

This LHR was then used in conjunction with the base rate presented (BR) to calculate trial by trial predicted posterior odds, calculated as:

$$Posterior\;Odds=\frac{BR\;}{1-BR}\;\times \;LHR$$

Finally, Posterior Odds were used in conjunction with E1 to calculate the degree to which a rational Bayesian agent would update on each trial, as:

$$Bayesian\;Update=\left|\frac{E1-Posterior\;Odds\;}{1\;+\;Posterior\;Odds}\;\right|$$

From here, two measures were calculated (Bayesian Difference, Bayesian Raito), both of which compare Bayesian Update with participants actual update (defined as above) observed:.

BayesianDifference=BayesianUpdate−Update

$$Bayesian\;Ratio=\frac{Update\;}{Bayesian\;Update}\;$$

Each of these measures were compared for upwards trials versus downwards trials using Wilcoxon paired difference test or paired sample *t* tests.

### Reinforcement learning

This analysis directly follows the procedure of Burton et al. which claims to follow a modelling approach presented by Kuzmanovic and Rigoux (Kuzmanovic et al., 2018; Kuzmanovic & Rigoux, 2017).

Updates (calculated as above) are modelled as:

Update=α×δ×(1−rP×w)

δ is a prediction error, defined as the difference between participants estimate of the base rate (eBR) and the actual base rate presented (BR):

δ=eBR−BR

*rP*—“relative personal knowledge”—is calculated according to whether estimates of the base rate are higher or lower than estimates of ones own likelihood, as:

$$\begin{array}{rl} & rp=\frac{eBR-E1}{eBR}\;if\;E1<eBR\\ & rp=\frac{E1-eBR}{100-eBR}\;if\;E1>eBR\\ & rp=0\;if\;E1=eBR \end{array}$$

α and w are free parameters. α, the learning rate, determines the degree to which beliefs change in proportion to the prediction error. w accounts for participants’ individual variability in their sensitivity to rP.

Rather than fit this model to participants updates to derive α and w estimates for each participant—which would be the normal approach for a reinforcement learning model of this form (Daw, 2011)—Burton et al. instead do the following.

First, they assume that w is 1 for all participants. This enables them to reduce the update equation to:

Update=α×δ×(1−rP)

Which in turn enables them to rearrange the terms of the Update equations such that α sits as the dependent variable:

$$\alpha =\frac{Update\;}{\delta \times (1-rP)}$$

Second, they use the above formulation to calculate a “trial by trial” learning rate (trials where update = 0, i.e. beliefs stay the same, the authors assume that α = 0). We note that this model specification is different to the one proposed by Kuzmanovic and Rigoux (in which a single learning rate is applied to *all* of a participants’ updates) and model comparison is used to compare different model specifications. We do not suggest others try to follow this approach, we are simply following Burton et al.’s flawed recipe.

α is then averaged for each participant for each condition (upwards, downwards) and then the two conditions compared using a Wilcoxon paired difference test.

### Details of Burton et al.’s experiments

For full details of the experiments conducted by Burton et al., we direct the reader to their paper. However, for convenience, we provide some brief details here. Burton et al. conduct 4 separate experiments. They use the same stimuli set and sample size (*N* = 100) in experiments 1–3. In experiment 4, Burton et al. used a subset (20/51) of the stimuli used in their experiments 1–3 and a larger sample size (*N* = 200). There are small differences in the design of the four experiments. Specifically, when in the experiment participants are asked to rate event valence and provide a revised self-estimate differs. In experiment 1, participants provided revised self-estimates in session 1 (after seeing the base rate for an event). In experiments 2–4, revised self-estimates were elicited in a separate session. Event ratings were either provided in a final session (experiments 1, 2 and 4) or in the first session at the end of each trial (experiment 3). The authors report only small variations in the results across studies and state these slight differences are not consequential. Participants were recruited from the online platform Prolific in all 4 experiments. Burton et al. opt to aggregate data over only 3 of the 4 experiments (not including data from the 4th experiment). A justification for this omission was not provided.

## Results

**Failure to find evidence of an update bias for neutral events in Burton et al. data.** Burton et al., conduct four experiments, each analysed using four approaches: Linear Mixed Models (LMM) (Marks & Baines, 2017), Bayesian analysis (Shah et al., 2016), Reinforcement Learning (Kuzmanovic & Rigoux, 2017) and the classic approach—Linear Regression (Kappes et al., 2018; Sharot et al., 2011). The three approaches which they report in the Supplementary Material—Bayesian, Reinforcement Learning and Linear Regression—all fail to show a bias in updating for neutral events (see Table 1). This includes the Bayesian ratio approach that the authors themselves advocated for previously (Shah et al., 2016).

**Table 1.** Belief update bias in neutral stimuli?

	Exp 1 (*N* = 100)	Exp 2 (*N* = 100)	Exp 3 (*N* = 100)	Exp 4 (*N* = 200)	Aggregate (*N* = 500)
Bayesian difference	Marginal (0.049)	Marginal (0.044)	Yes (0.013)	NO (0.22)	Yes (0.001)
Bayesian ratio	Yes (0.001)	NO (0.784)	NO (0.449)	NO (0.93)	NO (0.06)
Reinforcement learning	Yes (0.001)	NO (0.704)	NO (0.324)	NO (0.94)	NO (0.06)
Regression coefficient	Yes^a^ (0.001)	NO (0.662)	NO (0.540)	NO (0.28)	NO (0.07)

^a^ This specific effect holds only if the authors unique trial exclusion protocol is followed. This is a protocol that is bespoke to them and has not—to our knowledge—ever been followed by any other researchers using the Belief Update Task. If all trials are included (as would normally be the case), this effect also disappears (*t*(96) = 1.47, *p* = 0.15).

Burton et al.’s data reveals an effect of belief update bias for neutral events in study 1. This effect is highlighted in the title of their paper “Asymmetric Belief Updating Observed with Valence-Neutral Life Events”. Yet, they fail to replicate their own effect in studies 2, 3, 4 or in the aggregated data. *p* values are in parentheses.

**Failure to replicate Burton, Shah, Harris & Hahn.** We ran a new study (see Methods) in an attempt to replicate Burton et al. and failed to find any evidence of an update bias for neutral events. In particular there was no difference in the amount of updating in response to observing probabilities that were lower than expected relative to probabilities that were higher than expected. This failure was observed regardless of the analytic approach adopted.

Our study followed Burton et al.’s approach to the Belief Update Task but corrected for confounds they introduce, which are absent in the original task (Sharot & Garrett, 2022). All analysis was restricted to events participants rated as neutral (see Supplementary Table 1 for list of events used and their accompanying statistics).

First, we ran linear mixed effects models (LMMs), exactly as implemented by Burton et al. Update was entered as the dependent variable, direction of error (upwards/downwards) as the independent variable. Intercepts and slopes were taken as random effects (i.e. allowed to vary across participants). This revealed no difference in updating beliefs as a function of whether updating is upwards or downwards (F(1, 81.94) = 0.11, *p* = 0.74, Figure 1(c)). Even when re rerunning the model in a manner that inflates degrees of freedom (Barr et al., 2013; Judd et al., 2012; Murayama et al., 2014) by only including intercepts as random effects (as per the main analysis of Burton et al.), we still did not observe a bias in belief updating for neutral stimuli (F(1, 1918.7) = 0.68, *p* = 0.41). Finally, we reran the LMM excluding trials that could potentially be misclassified as upwards or downwards which can occur if the base rate presented sits between participants own estimate of the event occurring and their estimate of the base rate (Garrett & Sharot, 2017, 2014) (see Methods). Once again, this revealed no difference in updating beliefs as a function of whether updating is upwards or downwards (F(1, 86.29) = 1.05, *p* = 0.31).

Next, we turned to examine whether learning scores differed on trials when participants received numbers that are higher than expected vs. lower than expected. Learning scores are regression coefficients which express the degree to which participants are updating their beliefs in proportion to the error made. This is a “classic” approach used to analyse data from the task and that Burton et al. report in their Supplementary Material. Comparing these for downwards versus upwards once again revealed no difference in learning rates about neutral events, regardless of whether participants learned the event was more likely than anticipated or less likely (*t*(88) = 0.43, *p* = 0.67, paired sample ttest).

Burton et al., use three more analytic approaches. Two are Bayesian methods the authors have tried to popularise (Shah et al., 2016). The third is a Reinforcement Learning (RL) approach developed by Kuzmanovic and Rigoux (Kuzmanovic et al., 2018; Kuzmanovic & Rigoux, 2017). We note however that the belief updating task is not actually an RL task in which agents learn to select actions that maximise rewards iteratively via trial and error (Sutton & Barto, 2018). See for instance (Cazé & van der Meer, 2013; Lefebvre et al., 2017; Palminteri & Lebreton, 2022; Palminteri, Lefebvre, et al., 2017a) for biases in learning in RL. Burton et al.’s implementation is also at odds with what was actually proposed by Kuzmanovic and Rigoux (Kuzmanovic et al., 2018; Kuzmanovic & Rigoux, 2017). Namely, Burton et al. use an entirely different model specification in which learning rates change on a trial by trial basis (Courville et al., 2006; Pearce & Hall, 1980) and do not follow basic model fitting practices which would typically include parameter recovery, model comparison and out of sample prediction (Kuzmanovic et al., 2018; Kuzmanovic & Rigoux, 2017; Palminteri, Wyart, et al., 2017; Wilson & Collins, 2019).

Analysing the new data using these methods we find that two of these approaches reveal the *opposite* effect to that reported by Burton et al. (Bayesian ratio approach: median downwards = 0.46, median upwards = 0.67, *Z* = 3.07, *p* = 0.002, paired sample Wilcoxon test; Burton et al.,’s “Reinforcement Learning”; median downwards = 0.58, median upwards = 0.80, *Z* = 3.27, *p* = 0.001, paired sample Wilcoxon test). Only one approach—the Bayesian Difference approach—revealed an effect in the same direction as in Burton et al. (mean downwards = 0.08, mean upwards = 0.04, *t* = −2.99, *p* = 0.004, paired sample *t* test). In sum, we failed to replicate Burton et al.’s (2022) findings of belief update bias for neutral events.

## Discussion

The belief update task has been used by scientists around the world to test a wide range of questions related to belief formation and optimism (Kappes et al., 2018; Korn et al., 2016, 2012; Kube et al., 2022; Kuzmanovic et al., 2018, 2016, 2015; Kuzmanovic & Rigoux, 2017; Ma et al., 2016; Oganian et al., 2019). The central finding is that participants update their beliefs to a greater extent in response to desirable than undesirable information. For negative events (e.g. robbery) this means updating beliefs more when learning that the events are less likely than expected than more likely and for positive events (e.g. promotion) the other way around.

Burton et al., claim to show a similar pattern of belief updating in response to information about the likelihood of neutral events as has been previously shown in response to negative events. However, three out of four of their own experiments actually fail to show this effect in three of four analytic approaches they use. Moreover, they claim to show the effect over the aggregated data of the experiments. However, the authors actually aggregate their results over only three experiments despite conducting four separate experiments. When we re-examined their data, aggregating over all four experiments, we found that the aggregated results do not in fact show a bias in neutral events in three out of the four analytic approaches reported in their supplementary material, including the classic approach. Thus, the claim the authors repeatedly make in the manuscript—that their aggregated supplementary results show a belief update bias for neutral events—is misleading and demonstrably false.

Whilst reviewing the analysis code of Burton et al. in order to run this analysis, we found evidence that the researchers did initially aggregate data over all four experiments, but subsequently edited the relevant parts of the code to avoid incorporating the data from Experiment 4 in the final results they report. For example, in the code the authors used to compile the aggregate data for the Bayesian Ratio measure (available here,^1^ see code lines 562 and 613), the authors set up the data frame to compile the data with 500 rows (one row for each participant)—which is the participant count for all four experiments (there are 100 participants each in studies 1, 2, and 3, 200 participants in study 4). This results in 200 rows of missing data.

When we then examined the one analytic approach that shows an effect in Burton et al., we found that this is in fact only observed using LMMs that fail to include random effects (i.e. they only include a random intercept). Conducting statistical tests on a large number of data points which are “nested” within participants in this way (without accounting for this structure) incorrectly increases the power of the analyses. Indeed, it is well known that failure to incorporate random effects inflates degrees of freedom by 10–40 fold. This increases Type-1 error rates substantially and thus should not be reported (Barr et al., 2013; Judd et al., 2012; Murayama et al., 2014). Such errors have led to papers being retracted in the past (Fisher et al., 2015). The authors justify this approach as a model that includes random effects does not converge. However, this does not change the fact that they are inflating the likelihood of type-1 error by failing to include random effects. This inflation is why this specific analytic approach reveals significant findings while none of the other approaches do. We note that if one was to report LMMs that inflate degrees of freedom due to non-convergence, it would then be necessary to show that the same effect is observed using a different, statistically sound approach. In supplementary Tables S2–S6 they report additional LMMs that do not converge. The authors state they use LMMs because they wanted to follow a “precedent in this literature” but cite a solitary study—Marks and Baines (2017)—which also inflates degrees of freedom. Regardless, when we attempted to replicate Burton et al.’s results in a new study, we failed to do so even using LMMs that inflated degrees of freedom.

We also closely examined the task design Burton et al. used to collect their data. This revealed a number of confounds inserted into their version of the task, that are known to produce false results. Specifically, the authors took a task that has been carefully designed but changed key aspects of it including altering the response scale and skewing event probabilities (see Supplementary Figure 1). These changes introduce confounds that are well known (Garrett & Sharot, 2017; Sharot & Garrett, 2022), not least to the authors themselves who were previously criticised for generating spurious results in this way (Garrett & Sharot, 2017).

In the original task, very rare or very common events are not included—all event probabilities lie between 10% and 70%. Participants are told that the range of probabilities is between 3% and 77% and are only permitted to enter estimates within this range. This is done for two reasons. First, It is known that people’s perception of very low probabilities is distorted (Kahneman & Tversky, 1979). Second, it is important to ensure that the range of possible overestimation is equal to the range of possible underestimation. That is, if all event probabilities lie between 11% and 78% and participants are allowed to enter numbers between 0% and 100% then by design, they will not be able to update upwards as much as downwards. As a result, it has been established that if this paradigm is used to make claims about differences between downwards and upwards updating (regardless of whether the events are neutral, positive or negative), care has to be taken to use a set of base rates that are centred around the midpoint of the scale (Garrett & Sharot, 2017; Sharot & Garrett, 2022).

Burton et al., fail to do this in any of their experiments (see Supplementary Figure 1). The mean event base rate in their experiments sit close to 30% on a 0–100 scale. We have been very clear in the past (Garrett & Sharot, 2017; Sharot & Garrett, 2022) that such a large positive skew in the base rate distribution like this, will artificially create greater updating for “downwards trials” (where the base rate presented is lower than participants first estimate) compared to “upwards trials” (where the base rate presented is higher than participants first estimate), which is exactly the pattern observed by Burton et al. It is baffling why Burton et al. deliberately chose to test their hypothesis in four separate experiments using a scale and base rate set well-known to them to produce false positives.

Moreover, despite all past papers of the update bias including 20–40 trials per condition (that is per “good news” and “bad news”) the authors have on average seven trials per condition for neutral stimuli. They are thus increasing noise, which increases the likelihood of false findings (it is more likely that LMMs with random slopes included would have converged given a greater number of trials per condition). In addition, the authors fail to collect ratings of possible confounds which are always collected and controlled for when using the task (Garrett & Sharot, 2017; Ossola et al., 2020; Sharot et al., 2011; Sharot & Garrett, 2022). They claim this is because it has been shown that controlling for these variables does not change the results. This logic is flawed. The fact that the effect holds after confounds are controlled for when it is a true effect does not mean that it will hold when it is not a true effect (that is a bias for neutral stimuli). Thus, their results are unreliable and uninterpretable.

Given all of these concerns, we attempted to replicate Burton et al.’s findings by running a new study. We failed to find any evidence of a belief update bias for neutral events. In sum, we show that the claims made by Burton et al., are clearly not supported by their data, or anyone else’s.

## Supplementary Material

Supplemental Material

## Data Availability

Data from Burton et al. has been deposited (by Burton et al.) here: https://osf.io/8q74m/?view_only=9ea1dcb105164bda9f35228b3bb3495c. Data from the attempt to replicate study presented in this manuscript has been deposited here: https://osf.io/48v6z/?view_only=a045403ebb874287bdc66a95417bb597.

## Footnotes

1 https://osf.io/3nteq/?view_only=9ea1dcb105164bda9f35228b3bb3495c.
