# Supplementary material for: There is no belief update bias for neutral events: failure to replicate Burton et al. (2022)
Source: J Cogn Psychol (Hove). 2023 Aug 14;35(8):876–86. doi: 10.1080/20445911.2023.2245112 (PMC10591604; doi:10.1080/20445911.2023.2245112)
Supplement: Supplemental Material [file PECP_A_2245112_SM7073.docx]

**Supplementary Materials**

**Supplementary Figure 1.** Base rates used by Burton at al. for neutral events in each experiment. The mean base rate in each experiment sits well below 50, the midpoint of the response scale they opt to use, which was 0 to 100 (Experiment 1: t(1520)=-31.40, p<0.001; Experiment 2: t(1698)=-30.22, p<0.001; Experiment 3: t(1666)=-32.69, p<0.001; Experiment 4: t(1294)=-33.19, p<0.001, one sample ttests vs 50).


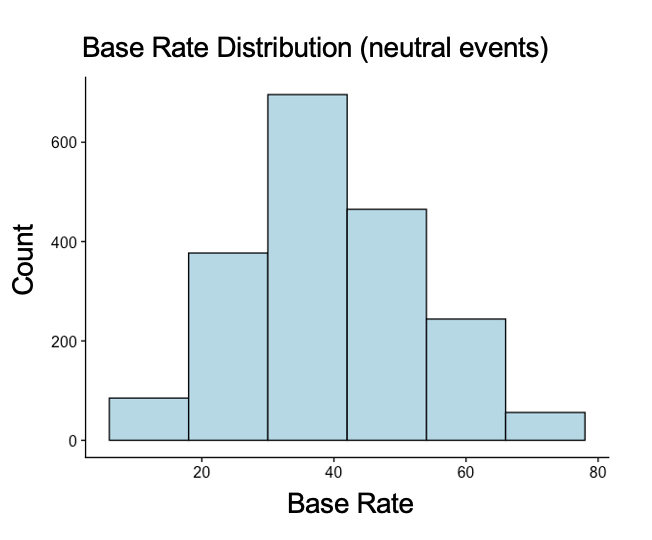


**Supplementary Figure 2.** Base rates for neutral events in the present study. The mean base rate in the experiment sits at the midpoint of the response scale which ranged from 10 to 70 (t(1922) = -1.20, p = 0.232, one sample ttests vs 40).

**Resampling Analysis**

Following a reviewer’s request, we conducted a reanalysis of Burton et al.’s data. Specifically, we resampled their data in order to try and generate a more normal distribution of base rates and then compared differences in update (downwards vs upwards) in this modified set of trials using LMMs again.

To do this, we only examined events rated neutral. Following Burton et al., mean updates in each of the two conditions (upwards/downwards) were calculated and outliers were removed (±3 × the interquartile range). We then deleted trials where base rates and estimates were less than 20% or above 80% (to compress the range of events towards the middle). Next, we assigned a probability of being sampled to each trial; trials with base rates between 45% and 70% had a 90% probability of being sampled. Trials outside of this range had a 10% probability of being sampled. Finally, we sampled with replacement the data over all subjects according to these assigned probabilities (the number of samples drawn was set equal to the size of the reduced dataset).

This procedure generated a more normally distributed set of base rates (see below for an example).


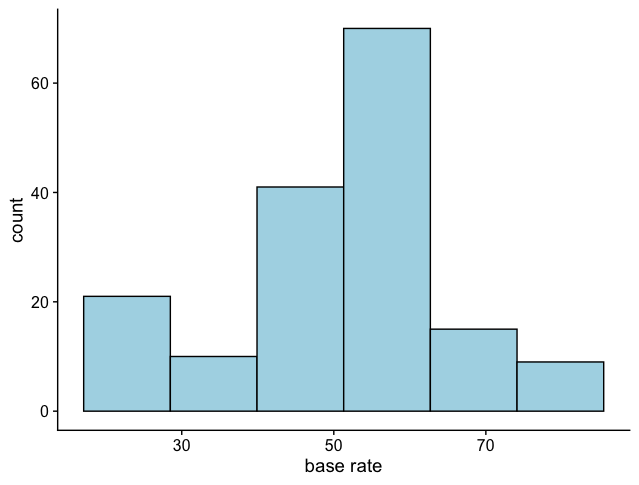


**Supplementary Figure 3.** Example Base Rate distribution following the resampling of Burton et al.’s data.

Next, we ran a Linear Mixed Model (LMM) on the resampled data with update as the dependent variable and direction (upwards/downwards) as the independent variable with intercept and slopes taken as random effects (i.e., update ~ direction + (1 + direction | Participant)). We ran this analysis 100 times (resampling the original data again each time using exactly the same procedure) for experiments 1, 2, 3 and 4 (i.e., 400 times in total). This resulted in the (artificial) bias of Burton et al. disappearing; a non-significant effect was found on 85%, 82%, 100% and 92% of these runs respectively. We note that on some runs, the LMM did not converge which may have led to inflated DFs. Thus, even the few runs that did produce significant results, should be taken with caution.

**List of Stimuli**

| **Event** | **Base Rate (BR)** | **Source** |
| --- | --- | --- |
| Meet with your supervisor in the next four weeks | 56 | Garrett & Sharot, 2017 |
| Participate in a game of sport in the next four weeks | 29 | Burton et al., 2022 |
| The next car that passes you is the colour black | 20 | Burton et al., 2022 |
| Use more than 3.7GB of mobile data over the next four weeks | 17 | Burton et al., 2022 |
| Meet your future spouse through an online dating service | 38 | Burton et al., 2022 |
| Marry someone with a different political affiliation to you | 26 | Burton et al., 2022 |
| The next person that you talk to has a positive impression of Golf cars | 62 | Webpage no longer available |
| The next person that you talk to has a positive impression of Ford Focus car | 59 | Webpage no longer available |
| The next new person you meet has a reduced ability to digest lactose | 65 | Webpage no longer available |
| The next email sent to you will be spam | 55 | <https://www.statista.com/statistics/270899/global-e-mail-spam-rate/> |
| The likelihood that you will receive less than 28 spam call in the next 4 weeks | 52 | <https://techcrunch.com/2020/12/07/spam-calls-grew-18-this-year-despite-the-global-pandemic/?guccounter=1&guce_referrer=aHR0cHM6Ly93d3cuZ29vZ2xlLmNvbS8&guce_referrer_sig=AQAAAL-epd69o2ik7B0KqAGYLjcKmZtyDMlkgyEm-v6sgnuE9md9-C8_dGLTYhY1NygVeGl0WXOcDlax4XgKENOo-BHSnDLHSt6iYDs13r4heTOx0AGZzGKt-LbuTRp39U822o7Pfqgz9PASadRpHg17PoA5XUB2AvvmHukjru5OBy35> |
| The next salesperson you see will have brown hair | 48 | <https://beachwaveperm.com/most-common-hair-color-in-uk/#:~:text=Report%20Ad-,2.,South%20and%20amongst%20indigenous%20Brits> |
| Have an extra artery in the arm | 30 | <https://www.sciencefocus.com/news/humans-are-evolving-an-extra-artery-in-the-arm/> |
| The next car you pass has been cleaned at least once in the past 3 months | 55 | <https://www.intelligentcarleasing.com/blog/how-clean-is-your-car-study/> |
| Buy laundry detergent in the next two weeks | 42 | <http://datacolada.org/22#footnote_0_574> |
| Download 1-3 new apps for your phone in the next month | 32 | <https://techcrunch.com/2017/08/25/majority-of-u-s-consumers-still-download-zero-apps-per-month-says-comscore/> |
| The next woman you walk past has a foot that measures (from heel to toe) 240mm or less | 41 | <https://www.nature.com/articles/s41598-019-55432-z.pdf> |
| Inhale and exhale 11,000 liters of air tomorrow | 48 | <https://www.sharecare.com/health/air-quality/oxygen-person-consume-a-day> |
| Use 90 gallons of water or less on a weekday next week | 49 | [https://www.usgs.gov/special-topic/water-science-school/science/water-qa-how-much-water-do-i-use-home-each-day?qt-science_center_objects=0#](https://www.usgs.gov/special-topic/water-science-school/science/water-qa-how-much-water-do-i-use-home-each-day?qt-science_center_objects=0) |
| The next stranger you see, walks at an average speed of 1.24 m/sec or less | 44 | <https://core.ac.uk/download/pdf/82088742.pdf> |
| The next stranger you walk past, walked between 6000 and 10000 steps the previous day | 33 | <https://www.researchgate.net/figure/Distribution-of-average-number-of-steps-per-day_tbl2_6747371> |
| The next stranger you walk past that owns a TV will watch 41 or less TV adverts tomorrow | 51 | <https://www.statista.com/statistics/486685/number-of-tv-ads-seen-daily-in-the-uk/> |
| The next stranger you walk past does not drink tea | 37 | <https://www.statista.com/chart/23081/most-consumed-drink-types-uk/> |
| The next student you pass in the street (aged 13-27) either types at a speed of 10-20 or 30-40 words per minute | 43 | <https://onlinetyping.org/blog/average-typing-speed.php#students> |
| The next stranger you walk past aged 65 or more does not have a smartphone | 39 | <https://www.statista.com/statistics/489255/percentage-of-us-smartphone-owners-by-age-group/> |
| The next stranger you walk past lives in a house with two, three or four other persons | 35 | <https://www.statista.com/statistics/281627/households-in-the-united-kingdom-uk-by-size/#statisticContainer> |
| The next adult male you meet has a body mass index between 18.5 and 24.9 | 30 | <https://researchbriefings.files.parliament.uk/documents/SN03336/SN03336.pdf> |
| The next woman you meet (aged 45-54) is 5ft 5inches tall or less | 70 | <https://allcountries.org/uscensus/230_cumulative_percent_distribution_of_population_by.html> |
| The next male you meet (aged 35-44) is 6ft 1inch tall or above | 10 | <https://allcountries.org/uscensus/230_cumulative_percent_distribution_of_population_by.html> |
| A prime number or a number less than 10 is drawn first in the UK national lottery this Saturday | 23 | [https://www.national-lottery.co.uk](https://www.national-lottery.co.uk/) |
| The first car you see next Monday has driven over 10,000 miles in the past year | 23 | <https://www.statista.com/statistics/513456/annual-mileage-of-motorists-in-the-united-kingdom-uk/> |
| The next adult male you meet drinks 5-7 cups of coffee per day | 36 | <https://www.researchgate.net/figure/Frequency-distribution-of-coffee-consumption-cups-day-at-age-32-years-and-its_tbl1_228615882> |
| The next song you hear is 210 seconds or less in duration | 33 | <http://theinformationdiet.blogspot.com/2011/11/probability-distribution-of-song-length.html> |
| The next stranger you pass in the street is less than 15 or older than 65 | 36 | <https://www.statista.com/statistics/270370/age-distribution-in-the-united-kingdom/> |
| Get a haircut in the next 4 weeks | 45 | Burton et al., 2022 |
| Drink between 56 and 84 cups of coffee over the next four weeks | 43 | Burton et al., 2022 |
| Yawn 6 times or less tomorrow | 38 | <https://reader.elsevier.com/reader/sd/pii/0031938495020144?token=0F37B34E4D887C55A3583F9FCAA6C508C641E69209FDFA6EA0C834EBBD60B87D13545017AA93044168AB22595708E9AB&originRegion=eu-west-1&originCreation=20211019115718> |
| The next 50 year old man you meet has an arm span of 173cm or less | 33 | <https://allcountries.org/uscensus/230_cumulative_percent_distribution_of_population_by.html> |
| Use 893 KwH of electricity (or more) in a month at least once in the next year | 47 | <https://www.eia.gov/tools/faqs/faq.php?id=97&t=3> |

**Supplementary Table 1.** List of events used along with their sources. These are normally distributed around a mean of 40 (the midpoint of the scale used).
